# Steps to improve the teaching of clinical reasoning in dermatology: A scoping review and proposal

**DOI:** 10.1002/ski2.352

**Published:** 2024-03-04

**Authors:** Aliya Rodriguez, Christopher Farkouh, Rebecca Raszewski, Claudia Hernandez

**Affiliations:** ^1^ Department of Dermatology Rush Medical College Chicago Illinois USA; ^2^ University of Illinois at Chicago University Library Chicago Illinois USA; ^3^ Section of Dermatology Jesse Brown VA Medical Center Chicago Illinois USA

## Abstract

Clinical reasoning (CR) is an area of active interest since faults in the diagnostic process can result in errors and possibly delays in care or even patient harm. The purpose of this scoping review was to collect information from the medical literature on approaches utilized to teach and assess CR in dermatology, identify gaps, and prepare a proposal on how to enhance the speciality's ability to develop trainee CR skills. We conducted a review of the published literature (1990–2020) from four databases. The initial search yielded 780 papers, and 42 relevant CR publications met inclusion criteria. Demographic, thematic content, theoretical frameworks, continuum of authenticity, competency/milestone, and assessment/educational intervention data were recorded by two screeners. Trainees at different educational levels from 17 different countries have been assessed in the dermatology literature. Most publications were of a single intervention, appeared underpowered, and had small sample sizes. Only two publications examined work‐based assessments (use real patients/stimuli). Knowledge‐based studies were the dominant theoretical framework with no studies exclusively focused on process‐based CR interventions. Simulation was well represented with 23 (55%) investigations. Rigorous studies that examine CR teaching and assessment in dermatology are lacking. Evidence‐based best practices for use of work‐based assessments, especially direct observation, need to be developed/adapted for dermatology and validated. Dermatology training programs would benefit from longitudinal data on trainee CR development, process‐based CR educational programs, metacognition CR exploration specific to skin disease diagnosis, and studies that yield practical recommendations on how to structure multi‐faceted assessments that assess CR.

## Graphical abstract

Clinical reasoning (CR) is an essential skill that all physicians must develop during their training, however little is known on how clinical reasoning is assessed in dermatology. A scoping review was performed to collect information from the medical literature on approaches utilized to teach and assess clinical reasoning in dermatology, identify gaps, and propose how to enhance the specialtys ability to develop and refine CR skills amongst trainees.

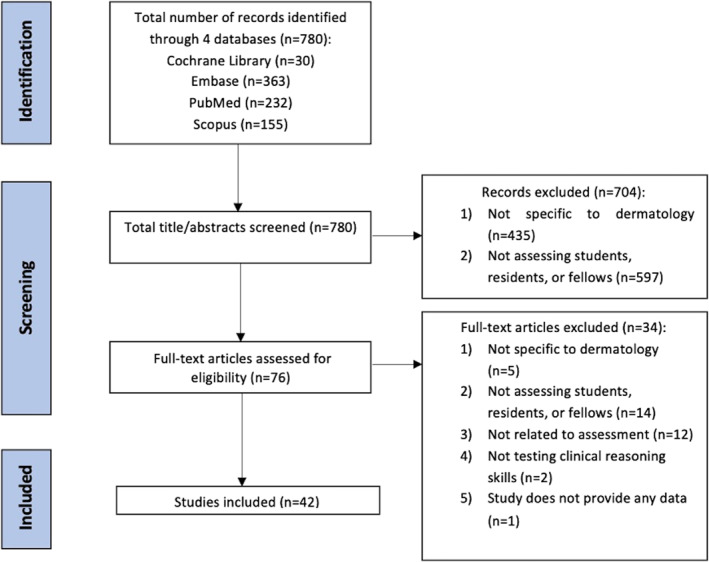

**What is already known?**

- The acquisition of clinical reasoning (CR) skills is a central objective in any medical training programme. However, little is known about how dermatology training programs are teaching and assessing CR. There are no standard guidelines on how to measure and improve CR acquisition amongst dermatology trainees.

**What does this study add?**

- This scoping review found that rigorous studies that examine CR in the teaching and assessment of trainees are lacking in the dermatology literature and offered proposals on how to enhance the speciality's ability to develop and assess CR skills.

## 1 INTRODUCTION

Critical to the achievement of medical competence is a poorly understood mental skill known as clinical reasoning (CR). Although definitions vary, CR is often described as ‘a skill, process, or outcome wherein clinicians observe, collect, and interpret data to diagnose and treat patients’.^1^ One of the central objectives of any residency programme is to develop accurate CR skills. Little is known about how dermatology residency programs (DRP) teach and assess CR.

Until recently, a multiple‐choice examination was utilized to evaluate dermatology graduates. This knowledge‐based exam heavily relied on memorization rather than scenarios requiring the application of medical knowledge to make accurate diagnostic and safe therapeutic decisions. In response, the American Board of Dermatology created the ‘applied’ exam where diagnostic and therapeutic decision‐making, both of which involve CR, is emphasized.^2^, ^3^ There is scant literature that can guide DRPs regarding the proper rate of CR development as well as standardized methods to measure CR acquisition of trainees throughout their training. Medical educators must continue to improve their understanding of CR development, how to accelerate its acquisition, and how to identify struggling learners early in their training and correct reasoning errors.^4^ The purpose of this scoping review was to collect information from the medical literature on the approaches utilized to teach and assess CR in dermatology, identify gaps, and prepare a proposal on how to enhance the specialty's ability to develop trainee CR skills.

## 2 BACKGROUND

Medical educators describe the challenge of CR research as trying to make the ‘invisible’ ‘visible’.^5^ A common starting point is to simplify CR by dividing it into a series of tasks. For example, Daniel et al.^6^ proposed seven CR tasks: (1) information gathering; (2) hypothesis generation; (3) problem representation; (4) generation of a differential diagnosis; (5) selection of a leading/working diagnosis; (6) diagnostic justification; and (7) development of management/treatment plan. Novices versus experts move with different levels of efficiency between steps.^6^ An ‘expert’ clinician appears to heavily rely on ‘intuitive, nonanalytic reasoning’ to rapidly generate an accurate diagnostic hypothesis.^7^ To achieve this level of expertise, Dreyfus and Dreyfus described a 5‐level staged progressive learning model that hinges on the student being exposed to progressive challenges in CR/skill scenarios.^8^ This forms the basis of a residency's experiential learning model since it is probable that experience plays an essential role in accurate problem representation.^9^ Some question whether CR can ever be truly separated from an individual's fund of knowledge/experience. In other words, are ‘experts’ simply in possession of a larger fund of knowledge? If amassing knowledge is the critical link then opportunities for deliberate practice should continue to form the backbone of training programs. Yet it seems clear that even when possessing vast knowledge, it has limits in its ability to explain CR skills. Expert clinicians also appear to have a superior ability to mentally organize increasingly intricate sets of clinical information for rapid retrieval without sacrificing diagnostic accuracy.^10^, ^11^, ^12^, ^13^ Efforts to understand the CR process have given rise to a number of conceptualizations that reflect different theoretical approaches and asssumptions about it.

A review of CR theories by Yazdani et al.^9^ divided them into three categories: (1) process‐based theories/models (T/M); (2) knowledge‐structure‐based T/M, and (3) compilation T/M. The first category (process‐based T/M) utilizes the hypothetico‐deductive model,^9^, ^10^, ^11^, ^12^, ^13^, ^14^ where CR is viewed as a largely sequential process with physicians generating a number of hypotheses that generate the basis for further enquiries to test them. The second category (knowledge‐structure T/M) utilizes illness script theory,^9^ where novices organize increasing amounts of knowledge into ‘illness scripts’.^15^ Scripts are list‐like mental structures with three components (predispoing conditions, pathophysiological insult, and clinical consequences). Clinicians incorporate additional factors into scripts and continually reorganize them creating greater ease of access when key history or clinical findings trigger memories.^11^, ^13^, ^16^ The third category (compilation T/M) utilizes either dual process or cognitive continuum theories.^9^ Dual process combines intuition, pattern recognition, as well as experience that are activated in System 1 to rapidly arrive at a diagnosis. Some complex diagnoses require a more analytic approach comparing previously constructed cases in a second process known as System 2, hence dual process.^17^ As a clinician gains greater experience, they can link new cases with previously constructed illness lists with greater efficiency and seamlessly shift, with minimal cognitive effort, been the two‐systems.^18^ A weakness of each of these 3 T/M is that they focus on one specific portion of CR and not the entire process. Most fail to account for external factors such as emotions, time available with patients, irrelevant signs/symptoms, and their impact on the processes used to reach a correct diagnosis.^18^, ^19^

What is found in the medical literature regarding methods used to teach and assess CR in dermatology? A better understanding of this process could lead to improvements in instruction as well as assessment methods that could accelerate the acquisition of CR among medical students, residents, and even practicing physicians. The scoping review approach was selected because it allows for exploration of current CR publications in dermatology, therefore allowing our team to identify important future CR projects for dermatology.

## 3 MATERIALS AND METHODS

The five‐stage methodological framework for scoping reviews published by Arksey and O’Malley in 2005^20^ was used for this review. The five steps are as follows: (1) identify the research question; (2) identify relevant studies; (3) select relevant studies; (4) chart the data; and (5) data summary and reporting.

The research team consisted of two medical students, a medical librarian, and the author. For step 2, the librarian performed subject and keyword searches based on the following concepts: dermatological or skin‐related disorders; medical educational groups such as students, residents, and fellows; patient simulation; and CR and examination methods. The following databases were used: Cochrane Library, Embase, PubMed, and Scopus. Inclusion criteria were: (a) peer‐reviewed journal articles, (b) original data, (c) from 1990 to 2020, (d) focused on CR interventions (either instruction/assessment, or discussions could be included), and (e) limited to dermatology. Exclusion criteria were: (a) non‐English language studies without an available translation; (b) book review and editorials; (c) studies not examining dermatology CR; (d) studies of health professionals who were not medical students, residents, or fellows, and; (e) dermatology theoretical CR literature reviews since they were often summaries of CR publications readily available in the medical education literature.

Data collection proceeded with two screeners performing narrative review and documenting the following information: author(s), title, publication year, study objective/aims, intervention, duration, study population, methods, outcome measures, results, analysis, major themes, assessed accrediation council for graduate medical education (ACGME) milestones, location (United States vs. other), work‐based assessment (WBA), non‐WBA, simulation, dermatology subject‐matter focus (dermatopathology, paediatric dermatology, general dermatology, procedural), and limitations.^21^ A 3rd screener reviewed all data files for accuracy and served as a ‘tie‐breaker’ for discordant findings. Microsoft Excel (Microsoft, Redmond) was used to faciltate descriptive data collection, analyses, and graphical presentations.

## 4 RESULTS

**FIGURE 1:**
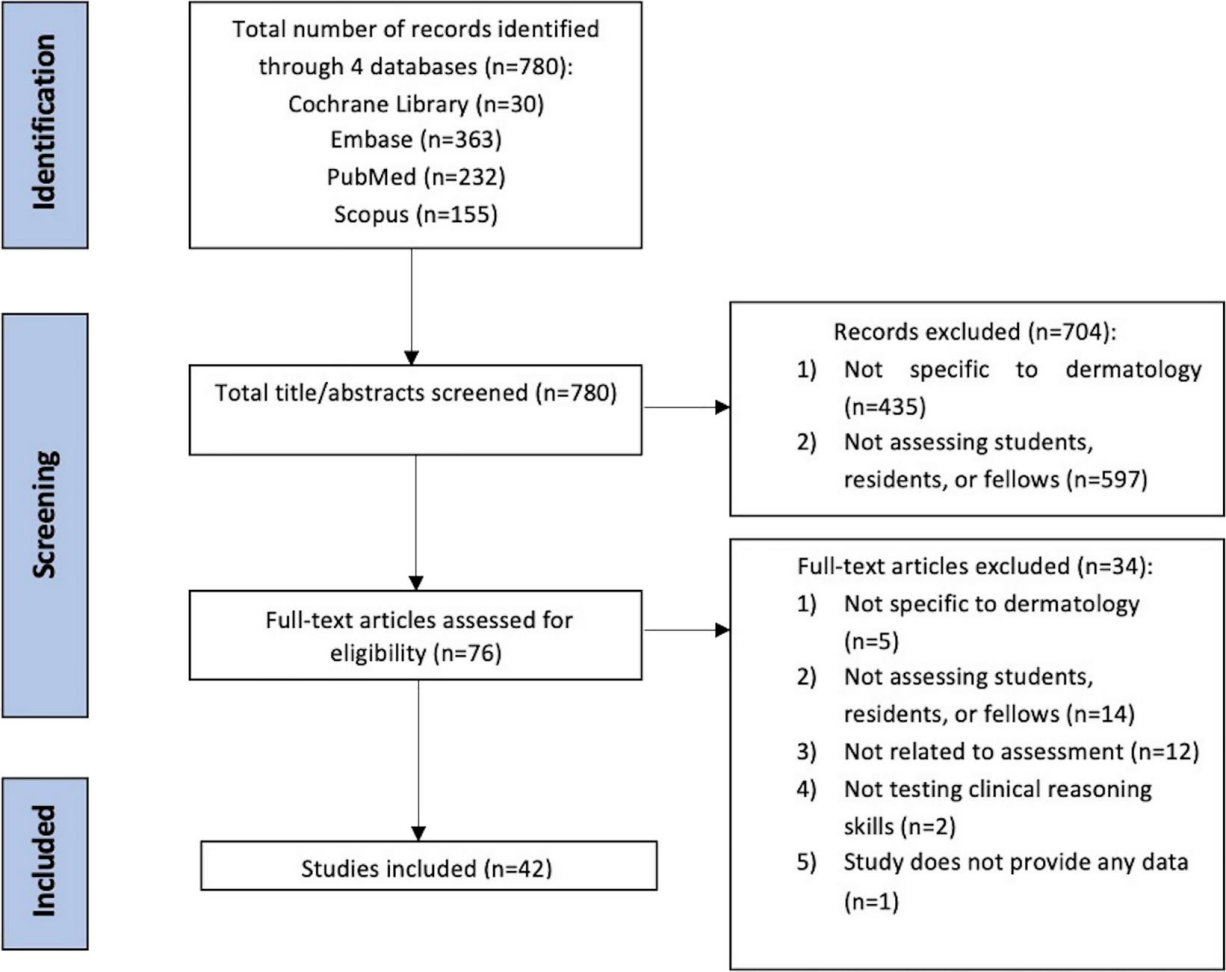
Preferred Reporting Items for Systematic Reviews and Meta‐Analyses flow diagram.

The initial search of the four databases yielded 780 papers as follows: Cochrane Library *n* = 30; Embase *n* = 363; PubMed *n* = 232; and Scopus *n* = 155. Of the screened manuscripts, 76 met initial inclusion criteria and upon further review, the selection was narrowed to 42 relevant publications (step 3) (Figure 1). Appendix S1 (see Supporting Information) contains a list of the 42 publications in alphabetical order. An overview of the included publications is provided in Tables 1 and 2. Our interrater concordance was 94%.

**TABLE 1:** Summary of articles.

First author (year); location	Population & sample size	Duration	Study aim	Dermatology focus area	Thematic content coding	Continuum of authenticity	ACGME^a^ milestones
Baker (2014); United States	First year medical students (*n* = 156)	3 years	To report student survey results of an activity that provides a realistic setting in which students perform skin biopsies and learn about skin histology.	Medical dermatology & dermatopathology	Decision making	WBA^b^	Patient care
Berger (2017); United States	Dermatology residents and pathology residents & fellows (*n* = 21)	Not specified	To determine whether examination scores would be equivalent when tested using two microscopy formats (glass slides & virtual).	Dermatopathology	Decision making	NWBA^c^	Patient care
Brick (2013); United States	Dermatology residents (*n* = 35)	1 academic year	To evaluate diagnostic accuracy and attitudes between virtual microscopy and glass slides.	Dermatopathology	Decision making	NWBA^c^	Patient care
Cervantes (2017); United States	First and second year medical students (*n* = 19)	1 month	To investigate the use of a cadaver to provide both cognitive and technical evaluations of skin biopsies.	Medical dermatology	Assess novel CR^d^ method; decision making	Simulation	Medical knowledge
Cervantes (2019); United States	First and second year medical students (*n* = 29)	2 weeks	To investigate the usefulness of computer‐based video instruction in dermatologic procedure training on a cadaver.	Procedural dermatology	Assess novel CR^d^ method	Simulation	Patient care
Chaudhary (2017); India	6^th^ semester medical students (*n* = 139)	6 months	To compare the effectiveness of computer assisted OSCE^e^ versus traditional OSCE^e^ as assessment tools for dermatology.	Medical dermatology	Assess novel CR^d^ method; assess existing CR^d^ method; decision making	Simulation	IPCS^f^; patient care; medical knowledge
Choi (2019); United States	Preclinical medical students (*n* = 30)	4 weeks	To evaluate the effectiveness of a technology‐based visual perception training system for melanoma detection with an image‐based assessment of retention.	Medical dermatology	Decision making	NWBA^c^	Medical knowledge
Cipriano (2013); United States	Fourth year medical students (*n* = 51)	2 weeks	To determine the impact of the integration of the American Academy of dermatology online curriculum into a 2‐week dermatology clerkship by comparing scores on MCQ^g^ test.	Medical dermatology	Course/curriculum (>1 session); decision making	NWBA^c^	Medical knowledge; patient care
Dietricha (2021); France	Dermatology residents (*n* = 15)	6 months	To investigate the benefits of simulation training in the disclosure of melanoma diagnosis or treatment.	Medical dermatology	Assess existing CR^d^ method	Simulation	IPCS^f^
El Miedany (2010); England	Dermatology trainees (*n* = 10)	Not specified	To assess the efficacy of a simulated problem‐based learning educational activity for scoring the psoriasis area and severity index.	Medical dermatology	Assess novel CR^d^ method; decision making	Simulation	Medical knowledge
Enk (2003); Israel	Sixth year medical students (*n* = 84)	9 months	To determine the efficacy of a 2‐week dermatology elective by administering Kodachrome slide‐based MCQ^g^ exam.	Medical dermatology	Course/curriculum (>1 session); decision making	NWBA^c^	Medical knowledge
Fox (2017); United States	Second year medical students (*n* = 396)	1 week	To determine the effectiveness of flipped classroom versus traditional classroom methodology by comparing MCQ^g^ final exam scores.	Medical dermatology	Course/curriculum (>1 session); decision making	NWBA^c^	Medical knowledge
Garg (2010); United States	Second year medical students (*n* = 90)	3 months	To determine the effectiveness of teaching with 3D prosthetic models of skin lesions versus 2D images by comparing MCQ^g^ exam scores.	Medical dermatology	Education class (1 session); decision making; assess existing CR^d^ method	NWBA^c^; simulation	Medical knowledge; patient care
Garg (2015); United States	Third year medical students (*n* = 179)	1 year	To develop an OSCE^e^ for the detection and evaluation of melanoma.	Medical dermatology	Decision making; assess existing CR^d^ method	Simulation	Medical knowledge; patient care
Goodyear (2005); United Kingdom	Senior hour officers (*n* = 14)	Not specified	To compare learning outcomes for a problem‐based learning course versus a traditional didactic course with an OSCE^e^ and MCQ^g^ exam.	Paediatric dermatology	Course/curriculum (>1 session); decision making	NWBA^c^; simulation	Medical knowledge; patient care
Goulart (2012); United States	Second year medical students (*n* = 59)	3 months	To evaluate recognition and response to a prosthetic melanoma placed on a standardized patient.	Medical dermatology	Decision making; assess existing CR^d^ method	NWBA^c^; simulation	Medical knowledge
Grover (2012); India	Sixth semester medical students (*n* = 71)	4 months	To develop a computer assisted OSCE^e^ as an assessment after completion of a dermatology rotation.	Medical dermatology	Decision making; assess novel CR^d^ method	Simulation	Medical knowledge; patient care
Hartmann (1998); United States	Second year medical students (*n* = 200)	Not specified	To develop interactive teaching mechanisms for a dermatology course and assess its effectiveness with a Kodachrome slide and MCQ^g^ exam.	Medical dermatology	Course/curriculum (>1 session); decision making	NWBA^c^	Medical knowledge
Hazan (2018); United States	Dermatology residents (*n* = 40)	3 years	To evaluate the impact of cadaver training sessions on improving surgical knowledge with pre‐ and post‐intervention quizzes.	Procedural dermatology	Decision making; assess novel CR^d^ method	Simulation	Patient care
Hernandez (2013); United States	Fourth year medical students (*n* = 190)	6 months	To examine the student's ability to detect melanomas using standardized patients and moulage.	Medical dermatology	Decision making; assess existing CR^d^ method	Simulation	Patient care
Jain (2013); United States	Third year medical students (*n* = 75)	2 years	To evaluate a melanoma simulation model to teach visual assessment and counselling skills using standardized patients and moulage.	Medical dermatology	Assess novel CR^d^ method; decision making	Simulation	Medical knowledge; patient care
Jenkins (2008); United States	Second year medical students (*n* = 73)	4 days	To assess the effectiveness of an online tutorial in teaching morphology and terminology in comparison to traditional lectures.	Medical dermatology	Decision making	NWBA^c^	Medical knowledge
Kaliyadan (2014); Saudi Arabia	Fifth year medical students (*n* = 129)	3 weeks	To validate the use of a computer based OSCE^e^ in a dermatology course combined with a written exam, case presentation, and professional behaviour assessment.	Medical dermatology	Decision making; assess novel CR^d^ method	WBA^b^; NWBA^c^; simulation	Medical knowledge; professionalism
Li (2013); China	Fourth year medical students (*n* = 120)	Not specified	To compare the impact of real patients, digital problem‐based learning, paper problem based‐learning, and traditional lecture‐based learning by administering a written exam and OSCE^e^.	Medical dermatology	Decision making	NWBA^c^; simulation	Medical knowledge; patient care
Liu (2019); United States	Dermatology residents (*n* = 31)	Not specified	To assess the impact of a video‐based, flipped classroom curriculum and surgical simulation skin models on procedural skills.	Procedural dermatology	Decision making; course/curriculum (>1 session)	Simulation	Patient care
Marsch (2014); United States	Dermatology and pathology residents (*n* = 31)	2 weeks	To determine the effectiveness of annotated digital pathology slides as a teaching tool by administering pre‐ and post‐tutorial MCQ^g^ quizzes.	Dermatopathology	Decision making	NWBA^c^	Patient care
Ochsendorf (2004); Germany	Medical students in sixth clinical term (*n* = 235)	2 consecutive clinical terms	To compare a problem‐oriented practical course to a bedside teaching course and standard practical course with pre‐ and post‐course MCQ^g^ exams.	Medical dermatology	Course/curriculum (>1 session); decision making	NWBA^c^	Medical knowledge
Ochsendorf (2006); Germany	Medical students in sixth clinical term (*n* = 231)	2 consecutive clinical terms	To determine whether a large group case‐based teaching approach combined with small group bedside teaching improves learning outcomes with pre‐ and post‐course MCQ^g^ exams.	Medical dermatology	Course/curriculum (>1 session); decision making	NWBA^c^	Medical knowledge
Pontius (2020); United States	Dermatology residents (*n* = 9)	1 academic year	To determine if there is a difference in knowledge acquisition between traditional and flipped classrooms with a MCQ^g^ quiz.	Medical dermatology	Course/curriculum (>1 session); decision making	NWBA^c^	Medical knowledge
Punj (2014); Australia	Sixth year medical students (*n* = 152)	Not specified	To investigate the value of tactile descriptive information of a lesion on the diagnostic accuracy of a pigmented skin lesion using MCQ^g^ tests.	Medical dermatology	Decision making	NWBA^c^	Medical knowledge; patient care
Reichel (2004); United States	Chief dermatology residents, residency programme directors, directors of dermatologic surgery (*n* = 95)	4 months	To assess how the surgical skills of dermatology residents are taught and evaluated within the United States through a survey.	Procedural dermatology	Descriptive	N/A	Patient care
Rimoin (2015); United States	Pre‐clerkship medical students (*n* = 236)	1 year	To investigate whether online perceptual and adaptive learning modules could efficiently train in skin lesion morphologies through MCQ^g^ tests.	Medical dermatology	Decision making	NWBA^c^	Medical knowledge
Robinson (1996); United States	Second‐ and fourth‐year medical students (*n* = 285)	2 years	To determine the likelihood of skin cancer detection with a standardized patient and a written exam.	Medical dermatology	Decision making; assess existing CR^d^ method	NWBA^c^; simulation	Medical knowledge; patient care
Sabzwari (2017); Pakistan	Third‐year medical students (*n* = 96)	1 day	To assess the validity and feasibility of moulage‐based simulation on standardized patients in summative assessment.	Medical dermatology	Decision making; assess existing CR^d^ method; education class	Simulation	Medical knowledge; patient care
Saceda‐Corraloa (2017); Spain	‘Sixth grade’ medical students (*n* = 28)	1 day	To determine the feasibility of implementation of a dermatology OSCE^e^ in medical school.	Medical dermatology	Decision making; assess existing CR^d^ method	Simulation	Medical knowledge; patient care; IPCS^f^
Smith (2009); United States	Third year medical students (*n* = 121)	1 year	To design a physical exam course and evaluate the course by developing an OSCE^e^ with a dermatology station.	Medical dermatology	Course/curriculum (>1 session); decision making	Simulation	Medical knowledge; patient care
Ternov (2020); Denmark	Medical students (*n* = 36) and physicians with 0–10 years of experience in general medicine, plastic surgery, or dermatology (*n* = 136)	3 months	To develop and gather validity evidence for a MCQ^g^ test for skin cancer diagnostics.	Medical dermatology	Decision making; assess existing CR^d^ method	NWBA^c^	Medical knowledge; patient care
Ulman (2015); United States	Fourth year medical students (*n* = 85)	1 day	To assess whether a medical school curriculum is adequately preparing medical students to diagnose and treat common dermatologic conditions through administering a MCQ^g^ quiz.	Medical dermatology	Course/curriculum (>1 session); decision making; assess existing CR^d^ method	NWBA^c^	Medical knowledge; patient care
Wahlgren (2006); Sweden	Medical students in the 7^th^ term (*n* = 116)	3 consecutive 17‐day courses	To develop an interactive educational dermatology computer programme for medical students and evaluate it with a final written examination.	Medical dermatology	Decision making	NWBA^c^	Medical knowledge; patient care
Waller (2019); Canada	Third year medical students (*n* = 250)	1 week	To develop and evaluate the results of a dermatology stations programme including standardized patients, simulated procedures, and MCQ^g^ exam.	Medical dermatology	Decision making	NWBA^c^; simulation	Medical knowledge
Wanat (2013); United tates	Second year medical students (*n* = 424)	3 years	To determine the effectiveness of a programme for teaching the total body skin exam using a standardized patient with moulage.	Medical dermatology	Decision making; assess existing CR^d^ method	Simulation	Patient care
Wang (2015); United States	Dermatology residents (*n* = 12)	Not specified	To pilot the use of a melanoma simulation model to improve standardization of clinical findings in a dermatology OSCE^e^ station.	Medical dermatology	Decision making; assess novel CR^d^ method	Simulation	Medical knowledge; patient care

Abbreviation: NWBA, Non‐work‐based assessment.

^a^ Accreditation council for graduate medical education.

^b^ Work‐based assessment.

^c^ Non‐work‐based assessment.

^d^ Clinical reasoning.

^e^ Objective structured clinical examination.

^f^ Interpersonal and communication skills.

^g^ Multiple‐choice question.

**TABLE 2:** Article demographics and characteristics.

Location	*n*
United States	26
Europe	8
India	2
China, Israel, Pakistan, Saudi Arabia, Australia, Canada	1 (each)

Study population	*n*
Medical students	31
Residents	11

Dermatology focus area	*n*
Medical dermatology	34
Procedural dermatology	4
Dermatopathology	4
Paediatric dermatology	1

Thematic content coding	*n*
Course/curriculum (>1 session)	11
Education class (1 session)	2
Decision‐making	39
Assess existing clinical reasoning method	12
Assess novel clinical reasoning method	9
Descriptive	1

Continuum of authenticity	*n*
Work‐based assessment	2
Non‐work‐based assessment	24
Simulation	23

Assessment of ACGME milestones	*n*
Medical knowledge	31
Patient care	27
Interpersonal and communication skills	3
Professionalism	1
PBLI^a^ and SBP^b^	0

^a^ Practice‐based learning and improvement.

^b^ Systems‐based practice.

### Demographics

Charting the data (step 4) began by collecting demographics. Studies from outside the U.S. complicated the review process due to differences in training rotations and duration. Despite this, we found trainees at different educational levels from 17 different countries that participated in dermatology CR studies. Eleven CR interventions utilized residents while 31 involved medical students. Except for a single publication, all interventions utilized a single academic year and/or class meaning no studies evaluated multi‐year CR progression among trainees or tried to determine performance targets by post‐graduate year. No publications examined dermatology faculty CR knowledge/skills in either teaching or assessment domains.

### Theoretical framework

Published dermatologic education interventions were categorized into knowledge‐versus process‐versus compilation‐based.^22^ The process‐oriented CR approach teaches learners how to reason via the use of clinical cases to breakdown student's reasoning by reviewing diagnostic algorithms, specific CR steps, or evaluating and ranking clinical information. Sessions are typically interactive and use a hypothetico‐deductive CR framework. Knowledge‐based CR interventions use a pathophysiological mechanistic approach that supports learning distinctions between similar or lookalike diseases. Compilation is a mixed analytic and non‐analytical approach. Unfortunately, most publications did not describe interventions in sufficient detail to categorize them with high accuracy however knowledge‐based interventions appear to be much more common. Since dermatology utilizes visual processes that rely on a non‐analytical approach, mixed approaches are likely underappreciated.

### Thematic content coding

The majority of published manuscripts focused on an area of medical dermatology; four focused on new dermatopathology technology (comparison of glass slides vs. digitized images), four interventions were in procedural dermatology (skill development in cadaveric labs), while only one manuscript concentrated on paediatric dermatology. Published manuscripts described the development of: (a) new course/curriculum (more than one session), *n* = 11; (b) new educational session (single session), *n* = 2; (c) decision‐making exercise or assessment, *n* = 39; (d) evaluation of an existing CR assessment method, *n* = 12; (e) development of a new CR assessment method, *n* = 9; or (f) descriptive publication, *n* = 1. A publication could have more than one theme. Of the relevant articles, 39 out of 42 utilized a decision‐making scenario, however, interventions focused on CR process results did not attempt to understand how data is integrated to reach a diagnosis. Research publications largely focused on improving education in one area of dermatology (i.e. melanoma diagnosis).

### Continuum of authenticity

Education interventions included computer‐based modules/flipped classrooms (*n* = 3), problem‐based learning (*n* = 3), simulated patients and cadaveric exercises, while interventions that focused on trainee assessment included computer‐based/online/CD‐ROM (*n* = 15), prosthetic/moulage (*n* = 9), images (*n* = 5), objective structured clinical examinations (*n* = 6), and cadaveric models (*n* = 3). Some publications used the same method (i.e. simulation) for both education and assessment of trainees resulting in mixed formats. Daniel et al.^6^ divided CR assessments into WBA, non‐WBA, and assessments in simulated environments (SIM) to categorize them along a ‘continuum of authenticity’. WBA are able to measure multiple components of CR in real clinical settings (high authenticity), followed by SIM, and non‐WBA (typically occurs in classroom).^6^ Non‐WBA (*n* = 24) were the most commonly used category in the dermatology literature likely due to their high internal consistency and ease of use across large numbers of learners. Their ability to assess CR is questioned since they are considered ‘part‐task’ due to their inability to capture the ‘whole’ CR process. Even less known is how these classroom‐based skills transfer to the clinical environment. SIM (*n* = 23) follows closely in the number of published studies and is valuable because SIM can assess multiple CR components. However, high‐fidelity simulations are expensive due to the resources needed to develop and administer them. Most published SIM (often using moulage) reports are small pilot studies making their eventual role in dermatology education unclear. WBA (*n* = 2) were vastly under‐represented despite their ability to assess multiple components of CR in authentic clinical settings.

### Assessment of ACGME core competencies/milestones

The ACGME milestones are patient care, medical knowledge, practice‐based learning and improvement (PBLI), interpersonal and communication skills (IPCS), professionalism, and systems‐based practice (SBP). Four milestones had assessments in the published dermatology literature: (1) medical knowledge, *n* = 31, (2) patient care, *n* = 27, (3) IPCS, *n* = 3, and (4) professionalism, *n* = 1. 33 interventions had a mixed data collection that included both medical knowledge and patient care outcomes. Research on the potential use of simulation as well as the development of WBAs could help address the lack of published milestone outcome data for PBLI or SBP and increase the rigour of the assessments for all milestones.

## 5 DISCUSSION

Scoping reviews explore the current literature with the goal of identifying gaps that point to potential future areas of research. Studies that examine practical CR targets for teaching and assessment among dermatology medical students/residents are lacking. Our scoping review also found a lack of published CR process‐oriented research. Published studies lack documentation on the use of CR algorithmic approaches, Bayes theorem (a theorem used in CR where the probability of a diagnosis is based on the disease prevalence and the sensitivity/specificity of the diagnostic test), distinction between pertinent positive and negative/normal findings, etc. when instructing trainees and instead appear to approach differential diagnosis from a knowledge‐structure standpoint by reviewing lists of similar diseases. This likely reflects a lack of clarity regarding the CR processes and/or its amorphous nature making it a challenge to know how to best approach teaching it.

Studies on dermatologic CR deserve a rigorous approach given our specialty's unique mix of medical and procedural patient care along with its reliance on visual recognition patterns. One possible starting point is to consider carrying out in‐depth interviews with recognized expert teachers to explore their thinking algorithms. In other words, dermatology should invest in metacognition exploration to better understand expert CR regarding skin disease. Creation of detailed algorithmic mapping of the integration of clinical data could help make dermatologic CR more concrete or visible to trainees. It is likely that experts approach complex diagnosis by performing mathematical probabilistic calculations when weighing data points. Increasing trainee comfort levels with this approach could improve diagnostic accuracy when pattern recognition is not sufficient to diagnose a patient. Another possible benefit to this approach is in the area of diagnostic error. Process‐oriented research could lead to a better understanding of where diagnostic errors begin to occur in skin disease diagnosis allowing faculty to improve diagnostic education exercises by targeting common errors and developing effective remediation programs.

Quantitative methods to evaluate diagnostic performance remain elusive in medicine and dermatology is no exception. More research is needed to help faculty utilize WBA given dermatology's dependence on outpatient clinics as its principal teaching environment. There are many challenges to assessing residents here including time, high patient volumes, and the nonsystematic nature of clinical practice resulting in over‐ and under‐representation of certain diseases. Association of Professors of Dermatology and American Academy of Dermatology could help develop programs to train faculty on the use of simple and inexpensive WBA CR education/assessment tools such as think alouds, charts recall, etc. and fund studies to develop effective specialty‐specific outpatient direct observation tools.

Faculty would also benefit from a robust framework that details how to best assess trainees and their expected clinical and procedural proficiencies at different points in their training. Dermatology educational research needs to increase study sample sizes so they are multi‐institutional and span then length of a residency programme in order to be able to provide longitudinal data with expected progressive developmental targets in regards to resident performance. Faculty should feel at ease choosing from several CR assessments in order to provide robust feedback to trainees.

Given the small size of many DRP, development of a national educational resource portal or toolkit could assist smaller residency programme faculty. A CR toolkit with critical materials such as expert recordings, discussion boards, and links to up‐to‐date resources could increase faculty comfort levels with some under‐utilized assessment tools such as WBAs. The goal of the toolkit could be to help faculty construct CR objectives as well as correctly choose and employ CR assessments. APD or AAD's web‐based platforms and annual meetings could represent an opportunity for hands on practice in regards to CR education and assessment. Establishing a means for faculty to learn and collaborate may advance CR understanding by providing a mechanism to increase sample size and expand the generalizability of future studies.

There are several limitations to our scoping review including access only to published data since internal training programme tracking mechanisms are not available for review. Our search string could have missed an important CR publication, and reviewing manuscripts required several months to screen meaning recent publications may have not been included in this review. Multi‐specialty interventions that included dermatology could have also been missed in the screening process. Lastly, screeners needed to make decisions/judgements about multiple components of studies that were not always explicitly defined by authors.

## 6 CONCLUSIONS

The importance of CR in the development and eventual skill set of a practicing physician cannot be understated. This review found that dermatology CR research is largely focused on trainee knowledge‐based exercises. Dermatology would benefit from longitudinal data on knowledge as well as process‐based CR programs, metacognition CR exploration specific to skin disease diagnosis, faculty development programs to increase the use of WBA, and studies that yield practical recommendations about how faculty should choose from a menu of CR tools to construct a multi‐faceted education and assessment programme.

## AUTHOR CONTRIBUTIONS

**Aliya Rodriguez**: Data curation (lead); formal analysis (lead); investigation (lead); writing – original draft (supporting); writing – review & editing (lead). **Christopher Farkouh**: Data curation (lead); formal analysis (lead); investigation (lead). **Rebecca Raszewski**: Data curation (lead). **Claudia Hernandez**: Conceptualization (lead); formal analysis (supporting); investigation (supporting); methodology (lead); supervision (lead); writing – original draft (lead); writing – review & editing (supporting)

## CONFLICT OF INTEREST STATEMENT

Claudia Hernandez, MD, MEHP is an editor at JAMA Dermatology. The other authors have no conflicts of interests to disclose.

## ETHICS STATEMENT

Not applicable.

## Supporting information

Supporting Information S1

## Data Availability

The data underlying this article will be shared on reasonable request to the corresponding author.
