# Supplementary material for: Steps to improve the teaching of clinical reasoning in dermatology: A scoping review and proposal
Source: Skin Health Dis. 2024 Mar 4;4(4):e352. doi: 10.1002/ski2.352 (PMC11297438; doi:10.1002/ski2.352)
Supplement: Supplementary file 1 — Supporting Information S1 [file SKI2-4-e352-s001.docx]

APPENDIX S1

Scoping review articles in alphabetical order:

1. Baker MG, Bradley EB, McCollum MA, Russell MA. The Cadaveric Skin Biopsy Project: description and student evaluation of an innovative approach to dermatology instruction in the preclerkship medical school curriculum. J Am Acad Derm. 2014; 71(2): 314-319. doi:10.1016/j.jaad.2014.02.022.

2. Berger WA, Townsend Kraft M, Murphy MJ, Elaba Z. (2017). Randomized comparison of virtual microscopy and glass microscopy among dermatology and pathology residents during a simulated in-training examination. J Cut Path. 2017; 44(4): 409-410. doi:10.1111/cup.12896.

3. Brick KE, Sluzevich JC, Cappel MA, DiCaudo DJ, Comfere NI, Wieland CN. Comparison of virtual microscopy and glass slide microscopy among dermatology residents during a simulated in-training examination. J Cut Path. 2013; 40(9): 807-811.

4.Cervantes JA, Costello CM, Maarouf M, McCrary HC, Zeitouni NC. (2017). The Use of a Fresh-Tissue Cadaver Model for the Instruction of Dermatological Procedures: A Laboratory Study for Training Medical Students. Dermatol Surg. 2017; 43(9): 1150-1156. doi:10.1097/dss.0000000000001179.

5 Cervantes J, Costello CM, Maarouf M, Kurtzman DJB, Shi VY. Computer-Based Video Instruction for Training Medical Students on Skin Biopsies. Derma Surg. 2019; 45(6): 811‐817. doi:10.1097/DSS.0000000000001670.

6. Chaudhary R, Grover C, Bhattacharya SN, Sharma A. (2017). Computer assisted Objective Structured Clinical Examination versus Objective Structured Clinical Examination in assessment of Dermatology undergraduate students. In J Derm Ven Lep. 2017; 83(4): 448-452.

7. Choi AW, Xu RS, Jacob S, Dulmage BO, Colavincenzo ML, Robinson JK, Xu S. (2019). Visual perception training: A prospective cohort trial of a novel, technology-based method to teach melanoma recognition. Postgrad Medl J. 2019; 95(1124): 350-352.

8. Cipriano SD, Dybbro E, Boscardin CK, Shinkai K, Berger TG. (2013). Online learning in a dermatology clerkship: piloting the new American Academy of Dermatology Medical Student Core Curriculum. J Am Acad Dermatol. 2013; 69(2): 267-272.

9. Dietrich E, Le Corre Y, Dupin N, Dréno B, Cartier I, Granry JC, Martin L. (2021). Benefits of simulation using standardized patients for training dermatology residents in breaking bad news. *Ann Dermatol Venereol*. doi:10.1016/j.annder.2020.11.003.

10. El Miedany Y, El Gaafary M, Youssef S, Almedany S, Palmer D. Using Simulation in Clinical Education: Psoriasis Area and Severity Index (PASI) Score Assessment. *Curr Rheum Rev. 2010; 12*(3): 195-201.

11. Enk CD, Gilead L, Smolovich I, Cohen R. Diagnostic performance and retention of acquired skills after dermatology elective. *Inter J Derm. 2003; 42*(10):, 812-815.

12. Fox J, Faber D, Pikarsky S, Zhang C, Riley R, Mechaber A, Kirsne, RS. (2017). Development of a Flipped Medical School Dermatology Module. *South Med J. 2017; 110*(5): 319-324.

13. Garg A, Haley HL, Hatem D. Modern moulage: evaluating the use of 3-dimensional prosthetic mimics in a dermatology teaching program for second-year medical students. *Arch Derm. 2010; 146*(2): 143‐146.

14. Garg A, Biello K, Hoot JW, Reddy SB, Wilson L, George PC. Integrated Skin Exam. The Skin Cancer Objective Structured Clinical Examination (SCOSCE): A multi-institutional collaboration to develop and validate a clinical skills assessment for melanoma. *J Amer Acad Derm.* 2015; 73(6): 959-965.

15. Goodyear HM. (2005). Problem based learning in a junior doctor teaching programme. *Arch Dis in Child.* 2005; 90(3), 275-278.

16. Goulart JM, Dusza S, Pillsbury A, Soriano RP, Halpern AC, Marghoob AA. (2012). Recognition of melanoma: A dermatologic clinical competency in medical student education. *J Amer Acad Derm. 2012; 67*(4): 606-611.

17. Grover C, Bhattacharya SN, Pandhi D, Singal A, Kumar P. Computer Assisted Objective Structured Clinical Examination: A useful tool for dermatology undergraduate assessment. *In J of Derm Ven Lepr.* 2012; *78*(4): 519.

18. Hartmann AC, Cruz Jr P D. Interactive mechanisms for teaching dermatology to medical students. *Arch Dermatol*. 1998; *134*(6): 725-728.

19. Hazan E, Torbeck R, Connolly D, Wang JV, Griffin T, Keller M, Trufant J. (2018). Cadaveric simulation for improving surgical training in dermatology. *Dermatol Online J.* 2018; *24*(6): 13030/qt5cq2n3vp

20. Hernandez C, Mermelstein R, Robinson JK, Yudkowsky R. (2013). Assessing students' ability to detect melanomas using standardized patients and moulage. *J Amer Acad Derm. 2013; 68*(3): e83-e88.

21. Jain N, Anderson MJ, Patel P, Blatt H, Davis L, Bierman J, Robinson JK. (2013). Melanoma simulation model: promoting opportunistic screening and patient counseling. *JAMA Derm.* 2013; 149(6): 710‐716.

22. Jenkins S, Goel R, Morrell DS. Computer-assisted instruction versus traditional lecture for medical student teaching of dermatology morphology: a randomized control trial. *J Am Acad Derm. 2008; 59*(2): 255‐259.

23. Kaliyadan, F., Khan, A. S., Kuruvilla, J., & Feroze, K. (2014). Validation of a computer based objective structured clinical examination in the assessment of undergraduate dermatology courses. *Indian J Dermatol Venereol Lepro. 2014; 80*(2): 134-136.

24. Li J, Li Q L, L, J, Chen ML, Xie HF, Li YP, Chen X. (2013). Comparison of three problem-based learning conditions (real patients, digital and paper) with lecture-based learning in a dermatology course: a prospective randomized study from China. *Med Teach.* 2013; 35(2): e963‐970.

25. Liu KJ, Tkachenko E, Waldman A, Boskovski MT, Hartman RI, Levin AA, Mostaghimi A. A video-based, flipped classroom, simulation curriculum for dermatologic surgery: A prospective, multi-institution study. *J Am Acad Dermatol. 2019; 81*(6): 1271-1276.

26. Marsch AF, Espiritu B, Groth J, Hutchens KA. The effectiveness of annotated (vs. non-annotated) digital pathology slides as a teaching tool during dermatology and pathology residencies. *J Cutan Pathol.* 2014; *41*(6): 513-518.

27. Ochsendorf FR, Boehncke WH, Böer A, Kaufmann, R. Prospective randomised comparison of traditional, personal bedside and problem-oriented practical dermatology courses. *Med Educ.* 2004; 38(6): 652‐658.

28. Ochsendorf, F. R., Boehncke, W. H., Sommerlad, M., & Kaufmann, R. (2006). Interactive large-group teaching in a dermatology course. *Med Teach.* 2006; *28*(8): 697-701.

29. Pontius LN, Hooten J, Lesesky E, Rao C, Nicholas M, Bialas R, Atwater AR. (2020). A comparison of knowledge acquisition and perceived efficacy of a traditional vs flipped classroom-based dermatology residency curriculum. *Cutis.* 2020; *105*(1): 36-39.

30. Punj P, Devitt PG, Coventry BJ, Whitfield RJ. (2014). Palpation as a useful diagnostic tool for skin lesions. *J Plast Reconstr Aesthet Surg.* 2014; *67*(6): 804-807.

31. Reichel JL, Peirson RP, Berg D. (2004). Teaching and evaluation of surgical skills in dermatology: Results of a survey. *Arch Derm. 2004; 140*(11):1365-1369.

32. Rimoin L, Altieri L, Craft N, Krasne S, Kellman PJ. Training pattern recognition of skin lesion morphology, configuration, and distribution. *J Amer Acad Derm*. 2015; 72(3): 489-495.

33. Robinson JK, McGaghie WC. (1996). Skin cancer detection in a clinical practice examination with standardized patients. *J Am Acad Dermatol.* 1996; *34*(4): 709-711.

34. Sabzwari SR, Afzal A, Nanji K. (2017). Mimicking rashes: Use of moulage technique in undergraduate assessment at the Aga Khan university, Karachi. *Education for health (Abingdon, England).* 2017; 30(1): 60-63.

35. Saceda-Corralo D, Fonda-Pascual P, Moreno-Arrones ÓM, Alegre-Sánchez A, Hermosa-Gelbard Á, Jiménez-Gómez N, Jaén-Olasolo P. (2017). Objective Structured Clinical Examination as an Assessment Tool for Clinical Skills in Dermatology. *Actas Dermo-Sifiliograficas. 2017; 108*(3): 237-243.

36. Smith MA, Burton WB, Mackay M. Development, impact, and measurement of enhanced physical diagnosis skills. *Adv Hth Sci Educ. 2009; 14*(4): 547‐556.

37. Ternov NK, Vestergaard T, Hölmich LR, Karmishol K, Wagenblast AL, Klyver H, Chakera AH. (2020). Reliable test of clinicians' mastery in skin cancer diagnostics. *Arch Dermatol Res*. 2020; doi:10.1007/s00403-020-02097-8.

38. Ulman, C. A., Binder, S. B., & Borges, N. J. (2015). Assessment of medical students' proficiency in dermatology: Are medical students adequately prepared to diagnose and treat common dermatologic conditions in the United States? *J Educ Eval Health Prof. 2015; 12*, 18. doi:10.3352/jeehp.2015.12.18

39. Wahlgren CF, Edelbring S, Fors U, Hindbeck H, Ståhle, M. Evaluation of an interactive case simulation system in dermatology and venereology for medical students. *BMC Med Educ. 2006;* 6: 40. doi:10.1186/1472-6920-6-40.

40. Waller BAM., Liu A, Fleming P, Lansang P. (2019). Undergraduate Dermatology Medical Education: Results of a Large-Scale Patient Viewing Program. *J Cutan Med Surg.* 2019; 23(5): 482-487.

41. Wanat KA, Kist J, Jambusaria-Pahlajani A, Lamarra D, Mackey A, Treat JR, Rosenbach, M. (2013). Improving students' ability to perform skin examinations and detect cutaneous malignancies using standardized patients and moulage. *J Amer Acad Derm.* 2013; *69*(5): 816-817.

42. Wang S, Seelaus R, Rea CA, Hernandez, C. (2015). Use of a melanoma simulation model in a dermatology objective structured clinical examination station. *Med Teach.* 2015; *37*(2): 202-203.
